# Extraction of Innate Immune Genes in Dairy Cattle and the Regulation of Their Expression in Early Embryos

**DOI:** 10.3390/genes15030372

**Published:** 2024-03-18

**Authors:** Xue Wang, Lili Guo, Wenguang Zhang

**Affiliations:** 1 College of Animal Science, Inner Mongolia Agricultural University, Hohhot 010018, China; wangxuer979293@163.com; 2 College of Life Science, Inner Mongolia Agricultural University, Hohhot 010018, China; 13474912747@163.com; 3 Inner Mongolia Engineering Research Center of Genomic Big Data for Agriculture, Hohhot 010018, China

**Keywords:** innate immune genes, comparative genomics, dairy cattle, early embryo, gene co-expression network

## Abstract

As more and more of the available genomic data have been published, several databases have been developed for deciphering early mammalian embryogenesis; however, less research has been conducted on the regulation of the expression of natural immunity genes during early embryonic development in dairy cows. To this end, we explored the regulatory mechanism of innate immunity genes at the whole-genome level. Based on comparative genomics, 1473 innate immunity genes in cattle were obtained by collecting the latest reports on human innate immunity genes and updated bovine genome data for comparison, and a preliminary database of bovine innate immunity genes was constructed. In order to determine the regulatory mechanism of innate immune genes in dairy cattle early embryos, we conducted weighted co-expression network analysis of the innate immune genes at different developmental stages of dairy cattle early embryos. The results showed that specific module-related genes were significantly enriched in the MAPK signaling pathway. Protein–protein interaction (PPI) analysis showed gene interactions in each specific module, and 10 of the highest connectivity genes were chosen as potential hub genes. Finally, combined with the results for differential expressed genes (DEGs), *ATF3*, *IL6*, *CD8A*, *CD69*, *CD86*, *HCK*, *ERBB3*, *LCK*, *ITGB2*, *LYN*, and *ERBB2* were identified as the key genes of innate immunity in dairy cattle early embryos. In conclusion, the bovine innate immunity gene set was determined and the co-expression network of innate immunity genes in the early embryonic stage of dairy cattle was constructed by comparing and analyzing the whole genome of bovines and humans. The findings in this study provide the basis for exploring the involvement and regulation of innate immune genes in the early embryonic development of dairy cattle.

## 1. Introduction

Researchers are carrying out extensive and meticulous studies on bovines, as the need for high-quality milk sources keeps increasing [1]. Animal husbandry involves significant research in the field of early mammalian development. Health during the gestation period and the health of the offspring depend on whether the embryo undergoes proper development successfully. The preimplantation development of mammals encompasses various stages, such as fertilization, cleavage, compaction, and the formation of blastocysts. During the initial phases of embryogenesis, the activation of the zygotic genome marks the primary instance of transcription in an organism’s life and plays a crucial role in the development of preimplantation mammalian embryos [2,3]. The timing of zygotic gene activation (ZGA) varies across species. In mice, ZGA happens at the 2-cell stage, whereas in pigs, cattle, goats, and humans, it occurs slightly later, specifically at the 4–8-cell stage [4,5,6]. Each organism controls the process of embryo formation by activating specific genes in a distinct way.

The transcriptome changes before and after ZGA are of great significance for studying the mechanism of embryonic development and in the search for key transcription factors. The transcriptome changes in innate immune genes during this period may also play an important role in regulating the homeostasis development of early embryos in the mother. It has been reported that innate immunity functions earlier than acquired immunity in the embryonic stage [7]. As research progresses, more and more researchers are focusing on the origin and evolutionary process of innate immunity genes in different species. Several studies have performed comparative genomic analyses of the TLR and TRIM families in mammals [8,9], but there is a lack of studies on mammalian innate immunity genomes. With the rapid development of genomic data, more and more of the available genomic data are being published, and several databases have been developed to decipher early embryogenesis in mammals [10,11,12,13]. For example, the DBTMEE and EMAGE database integrates gene expression data to study developmental patterns in mouse early embryos [10,11], EmExplorer contains transcriptome data for five species on the temporal activation of gene expression and 306 developmentally relevant pathways [12], and DevOmics stores multi-omics data on human and mouse early embryos and provides access to the data pathways and provides a variety of downstream functions, etc. [13]. However, these data sources do not indicate whether natural immune expression regulation is constructed and involved in early embryonic development in mammals, meaning that the relatively complete mammalian innate immune genome has not been updated in a timely manner, and studies of the transcriptional changes in innate immunity genes before and after ZGA are limited. Early mammalian embryos are highly sensitive to their local environment, but little is known about how embryos detect and respond to specific environmental cues, and the ability of embryos to detect and signal the presence of pathogens is unknown. Natural immunity is a broad defense system that provides the first line of defense against pathogen invasion, and it is present in almost all eukaryotes, relying on surface barriers and cells and proteins within the organism to achieve a wide range of defensive effects. Research on natural immunity has focused more on the defense against pathogens, and fewer studies have demonstrated the mechanism of natural immunity establishment in the early embryo. The aim of this study is to explore the spatial and temporal activation of natural immunity regulation during early embryonic development, by comparing the transcriptomes of early embryos in dairy cows (embryos obtained by in vitro fertilization, IVF), to identify the regulatory networks and candidate genes of innate immunity genes during early embryonic development, to explore the molecular markers of innate immunity during early embryonic development of cows, and to provide the basis for the improvement of the quality of dairy cattle embryos. In addition, it will provide a basis for improving the quality detection of cow embryos and provide reference data for improving the breeding marker-assisted management strategy and for improving the production efficiency of the animal husbandry industry.

## 2. Materials and Methods

### 2.1. Comparative Genomic Analysis

#### 2.1.1. Genome Data Query

Based on a published set of human innate immunity genes [14], a comparative genomic analysis of human innate immunity genes was performed with the bovine species (*B. taurus*). In this study, the CDS sequences, protein sequences, and annotation files for the 2 species were downloaded from the Ensembl website (for the above species, for genes with variable splicing, only the longest transcript of one gene was retained in these analyses; in addition, the total number of mRNA: total number of genes: total CDS sequences: total protein sequences = 1:1:1:1).

#### 2.1.2. Innate Immunity Gene Family Analysis

OrthoFinder has the capability to identify orthologous clusters and detect rooted gene trees for each of these orthologous clusters [15]. Using OrthoFinder v2.5.4 and Diamond software inference, the longest protein sequences of innate immunity genes for 1377 individuals were identified and used to determine bovine orthologous genes. Using the outcomes of gene family analysis, researchers identified a shared gene family and sequenced the protein sequence of the innate immunity genes in bovine species. The editable equations from the equation editor need to be inserted in a proper format.

#### 2.1.3. Identification of Collinearity of Innate Immunity Genes

Collinear segments refer to the homology of large segments within the same species or between two species, resulting from genome replication, chromosome replication, large segment replication, and species differentiation. The conserved sequencing of genes within homologous fragments means that they may also be functionally conserved. In this study, the JCVI (MCScan) v1.1.11 package in the Python software was used to identify the collinear gene pairs of innate immunity genes. Then, functional enrichment analysis was performed on all the collinear blocks, and the interaction of innate immunity gene networks in bovine collinear blocks was assessed by using the Metascape software v3.5 (http://metascape.org/gp/index.html#/main/step1. accessed on 20 December 2022).

### 2.2. Regulation of the Expression of Innate Immunity Genes in Early Embryos of Dairy Cattle

#### 2.2.1. RNA-Seq Data Processing

The early embryo transcriptome data from 12 dairy cows (2-cell stage: SRR14857183, SRR14857184; 8-cell stage: SRR14857186, SRR14857187, SRR14857188; morula stage: SRR14857190, SRR14857191, SRR14857192; blastocyst stage: SRR14857194, SRR14857195, SRR14857196) were downloaded from the NCBI’s High-Throughput Sequence Read Archive (SRA). All RNA-seq datasets were compared to reference genomes (Bos taurus ARS-UCD1.2). The genome localization information of the bovine reference genome was used to calculate the alignment between effective reads and gene regions. SAMtools v1.9 was used to sort the BAM-aligned files, generated from HISAT2 v2.2.1 by name [16]. The amount of gene expression was then calculated using featureCounts [17].

#### 2.2.2. Short Time-Series Expression Miner Analysis

A gene regulatory network is a complex and continuous dynamic system, and inter-gene regulation is a dynamic event that changes with a change of time and a change in the environment. By measuring gene expression at different time points in the cell cycle and under specific conditions, the gene expression data, that is, time-series gene expression data, contains rich gene regulation information. The STEM tool (Short Time-series Expression Miner) can be used to cluster, compare, and visualize gene expression data in short-time series (less than or equal to 8 time points). In the obtained cluster modules, the expression patterns with color were significant (*p* ≤ 0.05 after Bonferroni correction), and the expression patterns with the same color modules were similar. The input data was standardized in advance (log2FPKM). Therefore, select no normalization/add as 0, the STEM clustering method, the maximum number of model profiles as 20, and the maximum unit change in the model profiles between time points as 2.

#### 2.2.3. Innate Immunity Differentially Expressed Genes (DEGs) Analysis

Four stages of innate immunity DEGs (2 cells vs. 8 cells, 8 cells vs. morula, morula vs. blastula) were detected using DESeq2 v1.30.1 and default parameters in the R package [18]. The genes complied with the |log2FC| > 1 and the *p* < 0.05 standard resulted in genes being screened out as DEGs. The correlation between the expression of DEGs and the sample was calculated using bioinformatics (http://www.bioinformatics.com, accessed on 2 March 2023), and a heatmap was used to visualize the results.

#### 2.2.4. Weighted Gene Co-Expression Network Analysis

WGCNA (weighted gene co-expression network analysis) is a systems biology algorithm that describes the correlation between genes (co-expression relationship). WGCNA is used to find highly correlated gene modules (clustering), which can be outlined in terms of module characteristics or hub genes, and can relate the module to other modules or trait indicators of interest to explain scientific hypotheses [19].

In this study, we used 12 early embryo tissue samples from dairy cows, with a total of 1473 innate immunity expression genes, and FPKM data as input datasets for the co-expression network analysis.

The pickSoftThreshold function was used to calculate the soft threshold β parameter that met the distribution requirements of a scale-free gene co-expression network. After considering the stationarity of R^2^ and the mean connectivity, β = 5 was selected as the soft threshold of the natural immunity gene co-expression network at different stages in early embryos.

We specified a minimum gene total of 50 for the module, a MEDissThres of 0.1, with other parameters set as default and, in addition, we identified stage-specific modules with strong correlations between GS and MM values (*p* values < 0.05) and highly correlated module–trait relationships (correlation coefficients > 0.5).

#### 2.2.5. Functional Enrichment and PPI Analysis

For genes in specific modules, the Kyoto Encyclopedia of Genes and Genomes (KEGG) pathway and Gene Ontology (GO) analyses were conducted using the DAVID database (https://David.ncifcrf.gov, accessed on 14 March 2023). GO terms and pathways with a *p*-value < 0.05 were defined as significantly enriched. Genes were calculated using STRING, and the protein–protein interaction (PPI) network was obtained and imported into Cytoscape. The node sizes and colors indicated different node degrees, and the width of the edges indicated combined scores and intra-module connection weights.

## 3. Results

### 3.1. Establishment and Collinearity Analysis of Innate Immunity Gene Families

**Figure 1:**
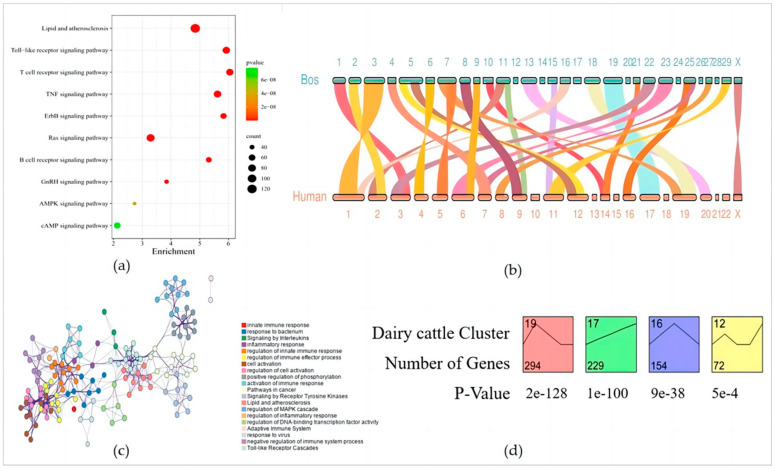
(**a**) The enrichment analysis of shared genes between the human and bovine species. (**b**) Bovine vs. human innate immunity gene collinear gene pairs. (**c**) Enrichment analysis of bovine collinear gene pairs. (**d**) Time−series modules of the genes.

In this study, 1377 human innate immunity gene protein sequences were used to perform all-vs-all sequence alignment of the input sequences using OrthoFinder, so as to determine the innate immunity gene protein sequences of other species. The protein sequences of 1473 innate immunity genes were obtained through analysis. A total of 2850 protein-coding genes from the two species were used for the analysis of the innate immunity gene families. Moreover, 2834 genes were identified in 1177 orthogroups, including 984 single-copy gene families. Overall, 1164 orthogroups were species common and 13 orthogroups were species specific (Table 1). In the common gene family, lipid and atherosclerosis, the toll-like receptor signaling pathway and the T cell receptor signaling pathway were significantly expressed, etc. (Figure 1a).

Genomic collinearity analysis is valuable for functional prediction of innate immunity collinear gene pairs, because genomic collinearity usually predicts homologous sequences, which may have similar functions. The innate immunity intergenomic collinearity was compared between bovines and humans using the JCVI software. It was found that there were 27 groups of collinear regions of innate immunity genes between bovines and humans, including 926 collinear gene pairs (Figure 1b). These innate immunity genes with collinear fragments accounted for about two-thirds of the total innate immunity genes in bovines.

According to the Metascape enrichment results, there was significant enrichment in the cytokine signaling in the immune system, positive regulation of cytokine production, regulation of kinase activity, regulation of the MAPK cascade, an adaptive immune system, a response to viruses, toll-like receptor cascades, and the immune response-regulating signaling pathway (Figure 1c). These pathways play a role in innate immunity, adaptive immunity, and inflammatory responses.

### 3.2. Trends in the Expression of Innate Immunity Genes in Early Embryos in Dairy Cattle

In order to explore the expression trend of innate immunity genes in early embryos, stem analysis was performed on the expression profile matrix of innate immunity genes in dairy cattle. A total of 1473 innate immunity genes in different stages of the early embryo in dairy cattle were analyzed and four significant modules were obtained, which were clustered into four categories (Figure 1d). This includes the NLRP gene family, among others.

Module 19, representing genes that have experienced an overall downward trend, indicated sustained degradation during the preimplantation period. This module contained 191 genes in covariate pairs, with the expression of NLRP14 and NLRP9, among others, peaking at the 2-cell stage and subsequently declining, suggesting that they may not be involved in zygotic development in dairy cattle. Module 17, representing the module whose expression increases with time, showed that this group of genes continued to rise during the preimplantation period, indicating their involvement in early embryonic development. This module contained 137 genes in collinear gene pairs, with PPIA and others peaking at the blastocyst stage, suggesting that they play a role in blastocyst formation and early differentiation. Module 16 represents the level of gene expression that first increases and then decreases. This module contained 96 collinear gene pairs, of which AKIRIN2 and NLRP8, for example, increased before the zygotic phase and decreased after the zygotic phase, indicating that these genes are involved in the activation of the zygotic genome only.

### 3.3. Construction of Weighted Gene Co-Expression Network and Module Detection

**Figure 2:**
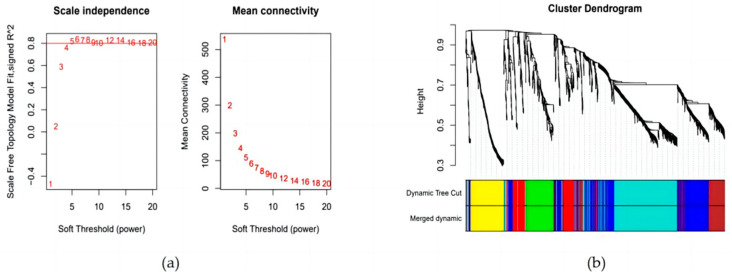
Scale independence and mean connectivity of co-expression network. (**a**) Screening of soft thresholds. (**b**) Cluster dendrogram.

Using WGCNA analysis, the relationship and function of innate immunity genes during the four embryonic development periods can be properly assessed. Based on scale independence and mean connectivity measures, the co-expression analysis in this study used 1473 innate immunity genes and confirmed that a soft threshold (β) = 5 and a scale-free network fitting index (R^2^) greater than 0.8 (Figure 2a) correspond to scale-free network features. The block module function was used to assign 1473 innate immunity genes to seven modules (Figure 2b), and the number of genes in each module varied significantly, from 123 genes in the red module to 341 genes in the blue module.

### 3.4. Identification of Specific Modules for Each Embryonic Stage of Development

**Figure 3:**
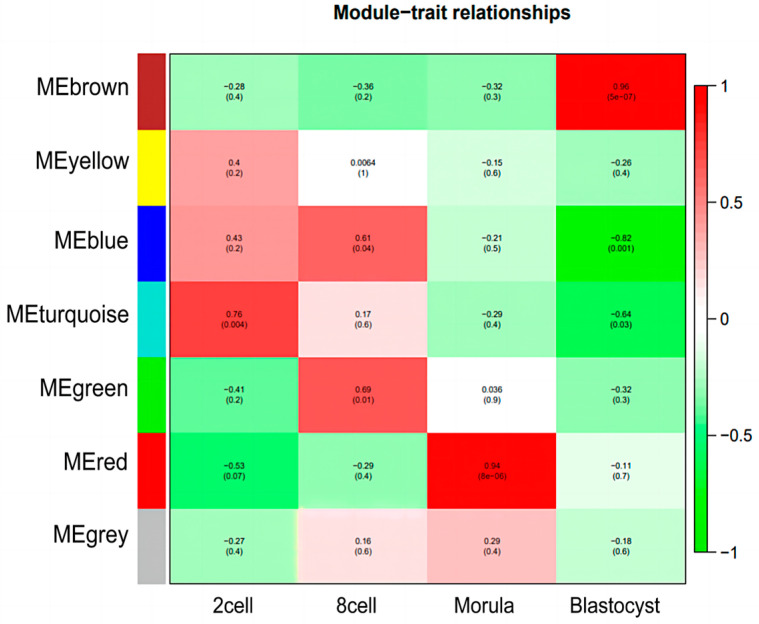
The correlation between the differentiation period and the modules. Red is positive and green is negative.

By calculating the GS (gene significance) and MM (module membership), two crucial metrics in the co-expression network analysis, the correlation between them is used to determine whether the genes associated with a trait play a significant role in the stage-specific module. As a result of using |R^2^| > 0.5 and *p* < 0.05 as the screening condition, four stage-specific modules were obtained, namely the turquoise module, blue module, green module, red module, and brown module, as shown in Figure 3. The turquoise module was positively correlated with the 2-cell stage (R^2^ = 0.76), the blue and green modules were positively correlated with the 8-cell stage (R^2^ = 0.61; R^2^ = 0.69), the red module was positively correlated with the morula stage (R^2^ = 0.94), and the brown modules were positively correlated with the blastocyst stage (R^2^ = 0.96).

### 3.5. Functional Enrichment of Stage-Specific Modules and Candidate Genes for Each Embryonic Stage of Development

GO and KEGG pathway enrichment analysis were carried out on the modules related to the early development of dairy cattle embryos, in order to better understand the biological functions of each module. After using DAVID enrichment, the items with significant enrichment were sorted according to the number of genes, and the top 10 items with a significant BP (biological process), CC (cell component), and MF (molecular function) were overlapped with other modules for analysis. As shown in Supplementary Figure S1, there is a rich overlap in biological functions in multiple modules, for example, zinc ion binding (turquoise, green, blue, and brown), the innate immune response (turquoise, green, blue, red, and brown), the inflammatory response (turquoise, green, blue, red, and brown), and cytoplasm (turquoise, green, blue, and brown). In addition, KEGG pathway analysis was performed using DAVID for modules significantly associated with early embryonic development in dairy cattle. The results show that most enrichment pathways overlap in several significantly related modules, such as NOD-like receptor signaling pathways (turquoise and red) and MAPK signaling pathways (turquoise, green, blue, and brown), indicating that innate immunity mechanisms are established in early embryos and regulate embryonic development. The toll-like receptor signaling pathway was the only enrichment pathway in the red module, indicating that toll-like receptors were more active in the morula period.

**Figure 4:**
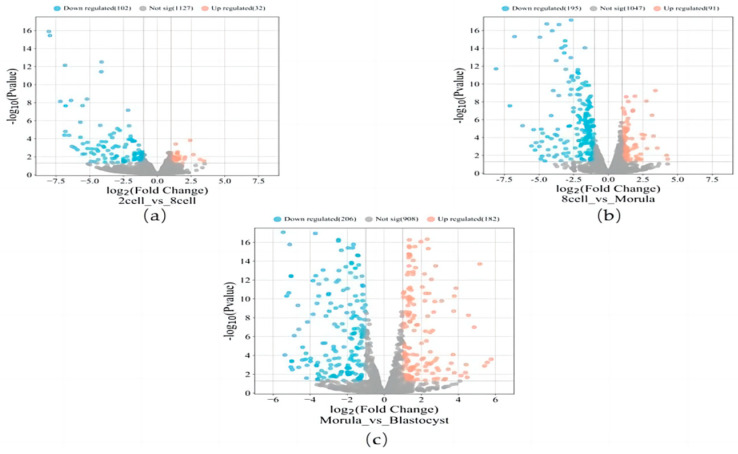
(**a**) Volcano plot for DEGs in 2-cell vs. 8-cell stage. (**b**) Volcano plot for DEGs in 8-cell vs. morula stage. (**c**) Volcano plot for DEGs in morula vs. blastocyst stage.

The differentially expressed genes were identified through pairwise comparisons between the four periods. In the 2-cell versus the 8-cell comparison, those with the least DEGs among the three comparisons, a total of 134 DEGs were observed, including 32 up-regulated genes and 102 down-regulated genes (Figure 4a). In a comparison of the 8-cell stage and morula stage, 286 DEGs were identified, including 91 up-regulated genes and 195 down-regulated genes (Figure 4b). The comparison between the morula and blastocyst stages showed the highest amount of DEGs, a total of 388 genes were identified, including 182 up-regulated genes and 206 down-regulated genes (Figure 4c). All of the above DEGs were jointly analyzed using the significance module in the WGCNA to determine the DEGs in the significance module.

**Figure 5:**
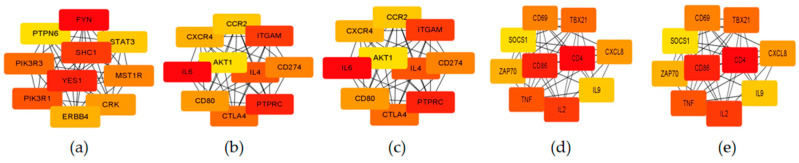
Visualization of potential genes in stage-specific modules. (**a**) PPI network for turquoise module. (**b**) PPI network for blue module. (**c**) PPI network for green module. (**d**) PPI network for red module. (**e**) PPI network for brown module. Different colors indicate the level of genetic connectivity, with red indicating high connectivity and yellow indicating low connectivity.

**Figure 6:**
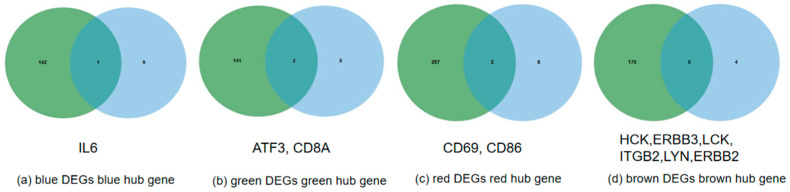
(**a**) Venn diagram of blue module DEGs and blue module hub gene. (**b**) Venn diagram of green module DEGs and green module hub gene. (**c**) Venn diagram of red module DEGs and red module hub gene. (**d**) Venn diagram of brown module DEGs and brown module hub gene.

**Figure 7:**
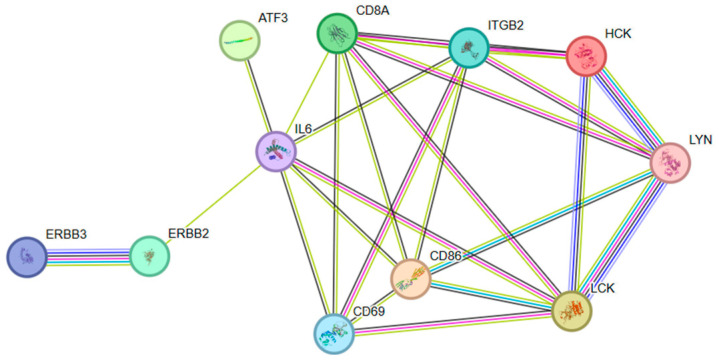
The network interactions between the 11 genes.

The PPI interactions between genes within the module were visualized using the cytoHubba plugin in Cytoscape, and the top 10 genes with the strongest connectivity were selected as candidate hub genes (Figure 5). Then, the intersection with DEGs was compared to determine the final key genes, namely ATF3, IL6, CD8A, CD69, CD86, HCK, ERBB3, LCK, ITGB2, LYN, and ERBB2 (Figure 6). Figure 7 shows the network interactions between the 11 genes.

## 4. Discussion

Mammals possess a remarkable ability to adapt to various environments and are found across diverse regions. They exhibit a high degree of diversity in terms of their habitat, behavior, and physical characteristics. However, when it comes to the innate immune system, they tend to display a strong inclination towards conservatism [20]. The continuous and fast-paced advancement in genomic data research has resulted in the increasing availability of published genomic data. However, this has led to a delay in updating the comprehensive genome of mammalian innate immunity. Hence, initially collating the most recent genetic data on human innate immunity, we proceeded to conduct a comprehensive comparison with the bovine genome. By employing the methodology of genome comparison, we have enhanced the precision and inclusivity of our investigation on bovine innate immunity genes. This will serve as a valuable reference for related research areas and helps to establish the groundwork for our forthcoming studies.

The initial phase of mammalian embryo development, distinct from the female oocyte stage, involves a transition from maternal to zygotic regulation. This transition involves the depletion of maternal factors and the activation of zygotic genes, ultimately resulting in the formation of totipotent embryos [21]. The continuous advancement of high-throughput sequencing technology has enabled broader and more in-depth exploration of the transcriptome of the early embryo. To determine the regulatory mechanisms of innate immunity genes during dairy cattle early embryonic development, we performed stem analysis and WGCNA analysis on dairy cattle early embryos. During the zygotic phase, NOD-like receptor and toll-like receptor signaling pathways exhibited sequential expression. Additionally, the MAPK signaling pathway was consistently enriched throughout all stages.

Using the comparative genome and published human innate immunity genes as a reference, we constructed the bovine innate immunity gene set and, subsequently, identified the corresponding collinear gene pairs in the innate immune system. The expression and co-expression network of genes related to the innate immune system in early embryos of dairy cattle were established following verification of the transcriptome of the early embryos. The trend of gene expression in innate immunity and the correlation between gene modules was explored, in order to understand the mechanism of transcription and gene expression in mammalian innate immunity. Eleven fundamental genes associated with the innate immune response in the early embryos of dairy cattle (*ATF3*, *IL6*, *CD8A*, *CD69*, *CD86*, *HCK*, *ERBB3*, *LCK*, *ITGB2*, *LYN*, *ERBB2*) were ultimately discovered, which are involved in the immune response, cell proliferation, and cell recognition.

NOD-like receptor signaling pathways have important roles in innate immunity and apoptosis. In recent years, specific NLRPs have been associated with reproduction and embryonic development and have been identified as maternal effect genes. It has been shown that NLRP genes are present at different embryonic stages [22,23,24]. The expression levels of NLRP 4, 5, 8, 9, 11, 12, 13, and 14 are higher in oocytes and, then, gradually decrease to very low levels in day-5 embryos. However, the expression levels of NLRP 2 and NLRP 7 showed different expression patterns in preimplantation embryos. Their expression pattern decreases to the lowest level on day 3 and, then, increases again on day 5 [25]. In addition, studies have also reported that specific NLRP genes play important roles in oogenesis and/or early embryonic development. Most of these reproductive studies have been conducted using mouse models and have provided new insights into the developmental roles of NLRP genes [26]. The toll-like receptor (TLR) family has been identified as a major family of pathogen recognition receptors (PRRs) [27,28] and mediates the innate immune response, which is the first line of defense for the host [29]. Wedad S et al. first reported on the expression and activity of many components of the innate immune TLR system in the human embryo. This is important for understanding the role of TLRs in preimplantation human development, which may be important for unraveling immunologic mechanisms and potential clinical markers of embryo quality and pregnancy initiation during natural conception and ART [30]. According to our analysis, NOD-like receptor signaling pathways and toll-like receptor signaling pathways were significantly enriched in early embryonic development in dairy cows, and the NOD-like receptor signaling pathway was specifically expressed during the 2-cell and morula stage, while the toll-like receptor signaling pathway was specifically expressed only during the morula stage, which suggests a temporal sequence in the construction of innate immunity mechanisms with different periods of early embryonic development, which are concurrently involved in the development of dairy cattle embryos.

The MAPK pathway is involved in a variety of cellular processes, including proliferation, differentiation, apoptosis, stress response, and inflammation. MAPK is a signaling cascade that includes various extracellular stimuli at the initiation of an inflammatory response, the production of pro-inflammatory cytokines, and their substrates [31]. The MAPK family consists of ERK1/2, ERK3/4, ERK5, ERK7/8, NLK, C-JUN, and p38, with two subgroups, namely classical MAPKs, such as ERK1/2, p38, JNK, and ERK5 and atypical MAPKs, such as ERK3, ERK4, ERK7, and NLK [32], and they are independent or interact with each other. ERK1/2 is induced in response to growth factors, hormones, and pro-inflammatory stimuli, while JNK1/2 and p38 MAPK are activated by cellular environmental stress and lead to inflammatory processes [33,34]. It is reported that a specific role for p38 MAPKs exists in providing a permissive translational environment during mouse blastocyst PrE differentiation [35]. Reports on the MAPK signaling pathway have focused more on cancer-related studies and less on its role in the early embryo. According to the results of WGCNA and pathway enrichment, most of the innate immune genes in dairy cattle are involved in the MAPK signaling pathway, and the MAPK signaling pathway is significantly enriched at all stages of the early embryo in dairy cattle, which implies that the early embryo in dairy cattle may secrete factors or proteins that participate in the recognition of the mother. At the same time, it may also mean that innate immunity genes in the early embryos of dairy cattle exercise functions beyond the scope of innate immunity mechanisms, and that they are more involved in cell division and differentiation; however, we need to further verify this idea in the future.

In addition, this study identified key genes associated with innate immunity in dairy cattle early embryos. *IL-6* is involved in the transition from innate to adaptive immunity by controlling leukocyte recruitment, activation, and apoptosis [36]. In recent years, it has been found that the *IL-6* cytokine family is involved in early embryonic development [37,38,39], and it is linked to the JAK/STAT 3 intracellular signaling pathway [40,41,42]. The JAK/STAT3 signaling pathway has been shown to be necessary for cell mass development within bovine embryos [43,44,45]. Another effect of *IL-6* is to preferentially promote the original endoderm (PE) cell lineage in IVP bovine blastocysts [46]. The role of *IL-6* in regulating inflammation is highly correlated with changes in uterine and trophoblastic attachment and implantation [47,48]. In addition, *IL-6* is recognized as an embryonic factor because *IL-6* increases the number of ICM cells in bovine blastocysts [49,50]. Our analysis showed that *IL-6*, as a key hub gene in the blue module, may be closely related to the development of dairy cattle at the zygotic phase, and that *IL-6* is interlinked with the other 10 genes obtained from the screening, constituting a regulatory network that is involved in early embryonic development in dairy cattle. This may also imply that the regulation of innate immunity genes at the zygotic phase influences the subsequent development of the early embryo in dairy cattle.

*ATF3* and *CD8A* are key hub genes in the green module. *ATF* encodes a member of the mammalian activating transcription factor/cAMP response element binding (CREB) protein family of transcription factors. It is induced by a variety of signals, including many encountered by cancer cells, and is involved in a complex process of cellular stress response [51]. *ATF3*-induced responses to a broad range of toll-like receptors (TLRs) have been observed, including TLRs 4, 2/6, 3, 5, 7, and 9, and, consequently, *ATF3* -/- primary macrophages exhibit increased production of *IL-6* and *IL-12* p40 cytokines upon TLR activation [52]. Gilchrist et al. reported that lipopolysaccharide (LPS) induced *ATF 3* -/- -*IL-6* and *IL-12* b mRNA levels in mouse bone marrow-derived macrophages (BMDMs). *ATF3* interacts with histone deacetylase 1, leading to inhibition of histone deacetylation, and *IL-6* and *IL-12* b promoter activity in LPS-treated macrophages. Thus, *ATF3* negatively regulates the transcription of proinflammatory cytokines containing ATF/CREB binding sites [53]. BMDMs show significantly lower survival after stimulation with multiple TLR ligands from *ATF3*-deficient mice compared with wild-type mice [54]. According to the WGCNA significance module and gene network interactions, both *IL6* and *ATF3* were significantly expressed during the 8-cell period and the toll-like receptor signaling pathway was significantly enriched only during the morula period, suggesting that the interactions between *IL6* and *ATF3* in the early embryonic development of dairy cows may affect toll-like receptor activity.

*CD8A* is a cell-surface glycoprotein that is present on most cytotoxic T lymphocytes and mediates effective cell–cell interactions within the immune system. CD8 antigens act as co-receptors with T cell receptors on T lymphocytes to recognize antigens displayed by antigen-presenting cells against the background of class I MHC molecules [55]. *CD8A*, as one of the CD8 molecular chains, is also involved in the immune response. In PRV-infected fish, high levels of *CD8A*, *CD8B*, and granzyme-A were found, suggesting that they have a positive regulatory effect on the CTL-mediated immune response [56].

*CD69* and *CD86* are key hub genes in the morula stage. *CD69* is a type II transmembrane glycoprotein belonging to the C-type lectin receptor family and a member of the NK cell signal transduction gene complex family. It is the earliest surface antigen expressed by T lymphocytes after activation and, when expressed, it can be used as a co-stimulatory signal to promote further activation and proliferation of T cells. *CD69* was also induced on NK cells, B cells, macrophages, neutrophils, and eosinophils [57,58]. *CD86* is expressed in dendritic cells, monocytes, memory T lymphocytes, germinal center B lymphocytes, activated B lymphocytes, and activated T lymphocytes. It belongs to the immunoglobulin superfamily and its ligands are CD28 and CD152 (CTLA4). *CD86* interacts with the inducer CD28 and the inhibitor CTLA4, which is the main cofactor in inducing T lymphocyte proliferation and IL-2 production [59]. NK cells, macrophages, neutrophils, eosinophils, dendritic cells, and monocytes are all innate immune cells. According to the enrichment results for the red module, the innate immune response has been expressed in this period, which means that *CD69* and *CD86* are secreted by innate immune cells to a greater extent, suggesting that *CD69* and *CD86* are involved in the construction of the innate immune mechanism of the early embryo in dairy cattle.

*ERBB2*, *ERBB3*, *LCK*, *LYN*, *HCK*, and *ITGB2* serve as key hub genes in the brown module, suggesting that innate immunity genes during the blastocyst period are more involved in the role of cell proliferation and differentiation and immune cell development. *ERBB2* and *ERBB3* are members of the epidermal growth factor receptor (EGFR) family that encodes receptor tyrosine kinases. This protein has no ligand-binding domain of its own and, therefore, cannot bind growth factors. However, it does bind tightly to other ligand-bound members in the EGF receptor family, forming heterodimers that stabilize ligand binding and enhance activation of kinase-mediated downstream signaling pathways, including the MAPK and PI3K signaling pathways [60]. Seiji Katagiri et al. showed that the normal expression profile of EGF affected the pregnancy rate of dairy cattle [61].

The Src kinase family is a group of proteins with tyrosine kinase activity, with a total of nine members, including *HCK*, *LCK*, and *LYN* [62]. Studies have shown that Src plays an important role in cellular processes, such as cell proliferation, differentiation, motility, and localization [63]. The proteins encoded by *HCK* are primarily involved in hematopoiesis, especially in bone marrow cells and B lymphoid lineage. It may help to pair Fc receptors with the activation of respiratory bursts. In addition, it may play a role in neutrophil migration and neutrophil degranulation [64]. *HCK* mediates the continuous activation and production of SATA5, and then activates downstream PI3K/AKT to promote cell growth and survival [65]. The protein encoded by *LCK* is a key signaling molecule in the selection and maturation of developing T cells [66]. Studies using *LCK* knockout mice, or *LCK* deficient T cell lines, have shown that *LCK* regulates the initiation of TCR signaling, T cell development, and T cell homeostasis [67]. *LYN* may be involved in mast cell degranulation and erythrocyte differentiation [68].

*ITGB2* encodes an integrin β chain, which binds to a number of different α chains to form different integrin heterodimers. Integrins are complete cell-surface proteins involved in cell adhesion and cell surface-mediated signaling. The encoded protein plays an important role in the immune response, and defects in this gene lead to defects in white blood cell adhesion [69].

What processes are innate immunity genes involved in, in the early embryo? Verena Ruprecht’s team published results in 2021 showing that even in the absence of specialized immune cells, early zebrafish and mouse embryos can recognize apoptotic cells through phosphatidylserine-mediated targets and effectively carry out phagocytic elimination of them [70]. Similar experimental results, and the results of our analysis, have led us to realize that innate immune genes may have a richer responsibility than “innate immunity”, and we hypothesized that in addition to the results obtained from this analysis, innate immune genes may also be responsible for the synthesis, transport, and supervision of intracellular substances, intercellular recognition, and the role of fate determination in cell differentiation during early embryonic development. At the beginning, scientists defined immunity as recognizing and killing foreign pathogens and, later, we thought that abnormal mutations in cells in the body could also be recognized and eliminated and, now, there is evidence to support the idea that early embryonic cells can recognize and eliminate programmed apoptosis cells; so, in the future, is it completely safe to assume that our cells, the recognition of the “self” and “others” in the earliest 2-cell period and the, subsequent, orderly division and differentiation may also be the result of the involvement of natural immunity genes. From the smallest units in plants and animals, to the single-celled life of natural eukaryotes, are the genes that we think are involved in innate immunity also the overseers and maintainers of order within their cells ?

While our research fails to address numerous bold assumptions, our findings have revealed previously unknown roles of innate immunity genes during early embryo development. Additionally, we have unraveled countless unexpected secrets associated with even the most basic processes, like DNA replication and cell division.

## 5. Conclusions

Using OrthoFinder sequence alignment analysis, researchers obtained a collection of 1473 genes related to innate immunity in bovines, which enabled them to create an initial set of bovine innate immunity genes. Subsequently, collinearity analysis was conducted to forecast homologous sequences, while enrichment analysis was executed to acquire pathways and interaction networks associated with the innate immunity of bovines. Through stem analysis, we acquired knowledge about the expression patterns of innate immunity genes during the early embryonic development of dairy cattle by combining it with transcriptome analysis. Utilizing WGCNA analysis, we constructed the gene co-expression network for various developmental phases of dairy cattle embryos, enabling us to uncover key hub genes associated with innate immunity during the early embryonic stage. By integrating findings from WGCNA and DEGs analysis, we have successfully identified *ATF3*, *IL6*, *CD8A*, *CD69*, *CD86*, *HCK*, *ERBB3*, *LCK*, *ITGB2*, *LYN*, and *ERBB2* as the pivotal genes for the innate immune response during the early development of dairy cattle embryos. The outcomes warrant additional validation and establish a theoretical foundation for the breeding of dairy cattle embryos.

## Figures and Tables

**Table 1:** Statistical results of innate immunity gene families.

Property	Value
Number of species	2
Number of genes	2850
Number of genes in orthogroups	2834
Number of unassigned genes	16
Percentage of genes in orthogroups	99.4
Percentage of unassigned genes	0.6
Number of orthogroups	1177
Number of species-specific orthogroups	13
Number of genes in species-specific orthogroups	66
Percentage of genes in species-specific orthogroups	2.3
Number of orthogroups with all species present	1164
Number of single-copy orthogroups	984

## Data Availability

All data are presented in the article.

## Supplementary Materials

The following supporting information can be downloaded at: https://www.mdpi.com/article/10.3390/genes15030372/s1, Figure S1. Results of GO analysis and KEGG enrichment in related specific module genes. (a) GO enrichment results of genes in turquoise module. (b) KEGG results of genes in turquoise module. (c) GO enrichment results of genes in blue module. (d) KEGG results of genes in blue module. (e) GO enrichment results of genes in green module. (f) KEGG results of genes in green module. (g) GO enrichment results of genes in red module. (h) KEGG results of genes in red module. (i) GO enrichment results of genes in brown module. (j) KEGG results of genes in brown module.
