# Supplementary material for: Extraction of Innate Immune Genes in Dairy Cattle and the Regulation of Their Expression in Early Embryos
Source: Genes (Basel). 2024 Mar 18;15(3):372. doi: 10.3390/genes15030372 (PMC10970270; doi:10.3390/genes15030372)
Supplement: Supplementary file 1 [file genes-15-00372-s001.zip › genes-2841968-supplementary.pdf]

Supplementary Figure S1

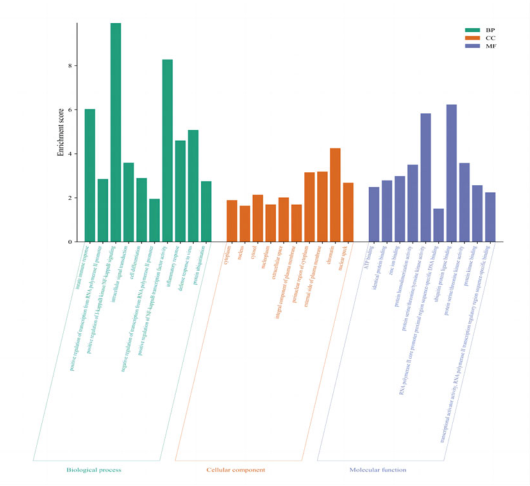

(a)

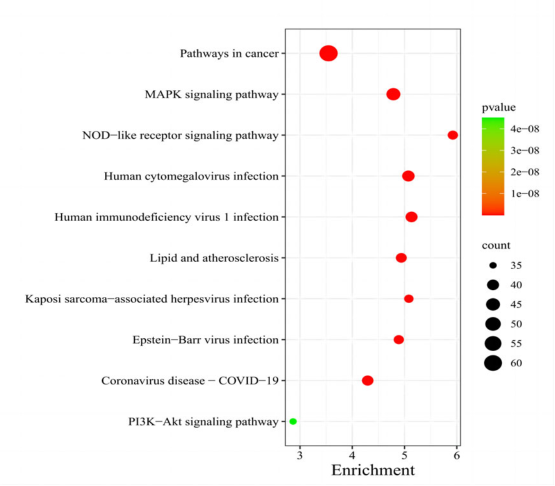

(b)

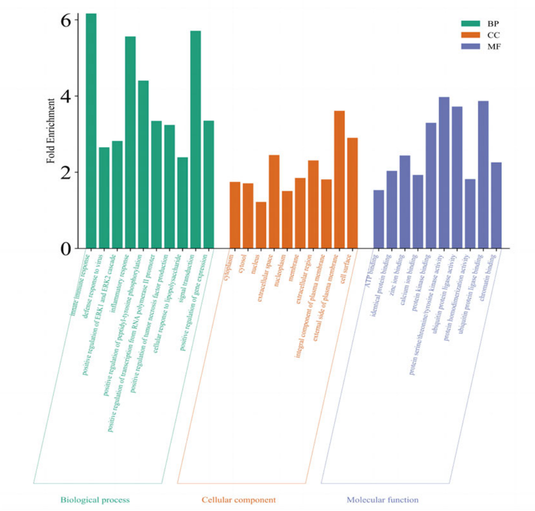

(c)

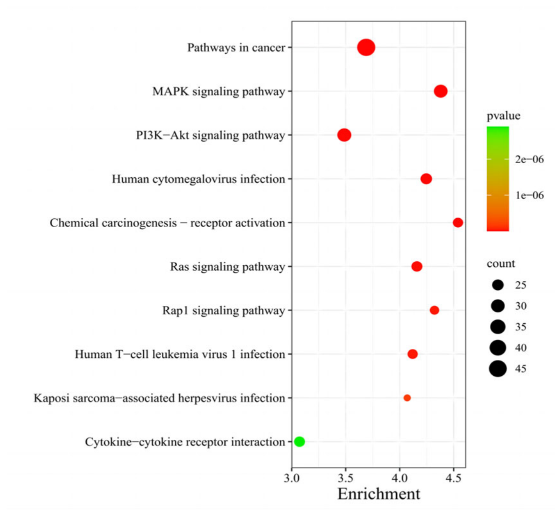

(d)

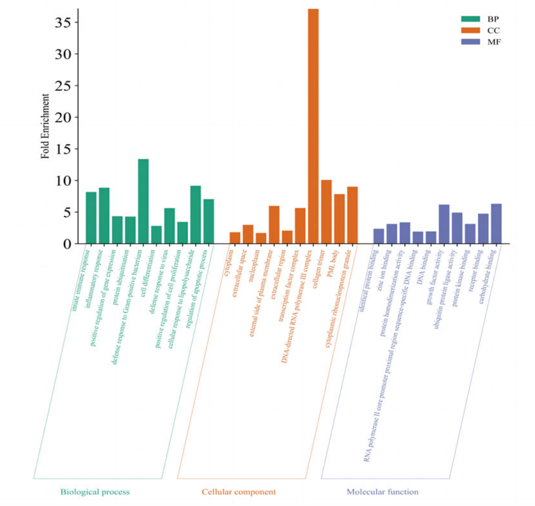

(e)

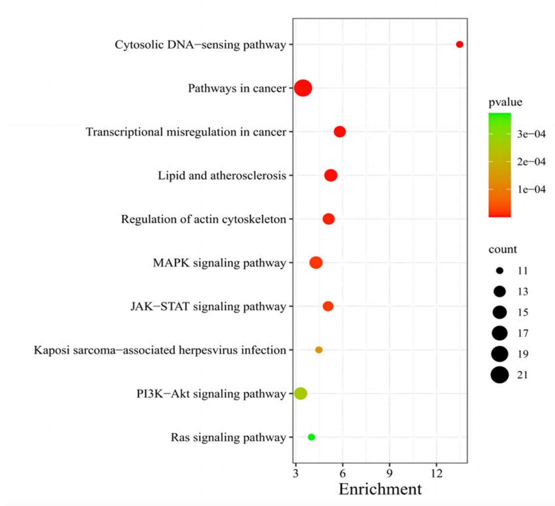

(f)

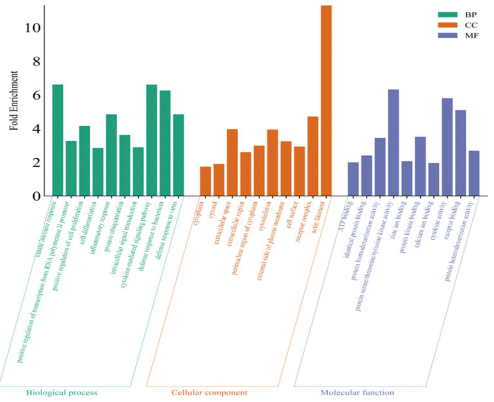

(g)

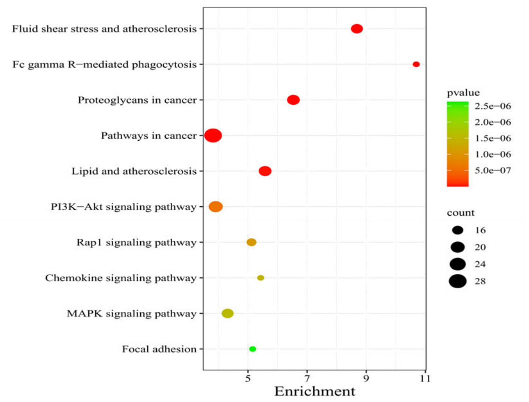

(h)

Supplementary Figure S1. Results of GO analysis and KEGG enrichment in related specific module genes. (a) GO enrichment results of genes in turquoise module. (b) KEGG results of genes in turquoise module. (c) GO enrichment results of genes in blue module. (d) KEGG results of genes in blue module. (e) GO enrichment results of genes in green module. (f) KEGG results of genes in green module. (g) GO enrichment results of genes in red module. (h) KEGG results of genes in red module. (i) GO enrichment results of genes in brown module. (j) KEGG results of genes in brown module.
